# Urinary sodium-to-potassium ratio associates with hypertension and current disease activity in patients with rheumatoid arthritis: a cross-sectional study

**DOI:** 10.1186/s13075-021-02479-x

**Published:** 2021-03-27

**Authors:** Hiroto Minamino, Masao Katsushima, Motomu Hashimoto, Yoshihito Fujita, Tamami Yoshida, Kaori Ikeda, Nozomi Isomura, Yasuo Oguri, Wataru Yamamoto, Ryu Watanabe, Kosaku Murakami, Koichi Murata, Kohei Nishitani, Masao Tanaka, Hiromu Ito, Koichiro Ohmura, Shuichi Matsuda, Nobuya Inagaki, Akio Morinobu

**Affiliations:** 1 Department of Diabetes, Endocrinology and Nutrition, Graduate School of Medicine, Kyoto University, 54 Shogoin, Kawahara-cho, Sakyo-ku, Kyoto-shi, Kyoto, 606-8507 Japan; 2 Japan Society for the Promotion of Science, 5-3-1 Kojimachi, Chiyoda-ku, Tokyo, 102-0083 Japan; 3 Department of Rheumatology and Clinical Immunology, Graduate School of Medicine, Kyoto University, 54 Shogoin, Kawahara-cho, Sakyo-ku, Kyoto-shi, Kyoto, 606-8507 Japan; 4 Department of Advanced Medicine for Rheumatic Diseases, Graduate School of Medicine, Kyoto University, 54 Shogoin, Kawahara-cho, Sakyo-ku, Kyoto-shi, Kyoto, 606-8507 Japan; 5 Department of Epidemiology for Community Health and Medicine, Kyoto Prefectural University of Medicine, 465 Kajii-cho, Kamigyo-ku, Kyoto, Kyoto-shi, Kyoto, 602-8566 Japan; 6 Department of Health Information Management, Kurashiki Sweet Hospital, 3542-1 Nakasho, Krashiki, Okayama, 710-0016 Japan; 7 Department of Orthopaedic Surgery, Graduate School of Medicine, Kyoto University, 54 Shogoin, Kawahara-cho, Sakyo-ku, Kyoto-shi, Kyoto, 606-8507 Japan

**Keywords:** Urinary sodium-to-potassium ratio, Rheumatoid arthritis, Disease activity, Blood pressure, Hypertension

## Abstract

**Background:**

Excessive salt intake is thought to exacerbate both development of hypertension and autoimmune diseases in animal models, but the clinical impact of excessive salt in rheumatoid arthritis (RA) patients is still unknown. We performed a cross-sectional study to clarify the associations between salt load index (urinary sodium-to-potassium ratio (Na/K ratio)), current disease activity, and hypertension in an RA population.

**Methods:**

Three hundred thirty-six participants from our cohort database (KURAMA) were enrolled. We used the spot urine Na/K ratio as a simplified index of salt loading and used the 28-Joint RA Disease Activity Score (DAS28-ESR) as an indicator of current RA disease activity. Using these indicators, we evaluated statistical associations between urinary Na/K ratio, DAS28-ESR, and prevalence of hypertension.

**Results:**

Urinary Na/K ratio was positively associated with measured systolic and diastolic blood pressure and also with prevalence of hypertension even after covariate adjustment (OR 1.34, *p* <  0.001). In addition, increased urinary Na/K ratio was significantly and positively correlated with DAS28-ESR in multiple regression analysis (estimate 0.12, *p* <  0.001), as was also the case in gender-separated and prednisolone-separated sub-analyses.

**Conclusion:**

Urinary Na/K ratio was independently associated with current disease activity as well as with prevalence of hypertension in RA patients. Thus, dietary modifications such as salt restriction and potassium supplementation should be investigated as a potential candidate for attenuating both disease activity and hypertension in RA patients.

**Supplementary Information:**

The online version contains supplementary material available at 10.1186/s13075-021-02479-x.

## Background

Rheumatoid arthritis (RA) is a chronic autoimmune disease characterized by articular destruction and increased risk of comorbidity and mortality [1]. Over the past decades, clinical outcomes of RA have been dramatically improved by new therapeutics such as biological disease-modifying antirheumatic drugs (bDMARDs) and Janus kinase (JAK) inhibitors [2, 3]. Despite such therapy, some patients continue to exhibit sustained high disease activity, which suggests involvement of unknown genetic or environmental factors.

A number of studies have reported that environmental factors participate in the pathogenesis of RA, including smoking, poor dental care, microbial imbalance, and poor dietary habits [4, 5]. Recently, in experimental animal models, excessive salt intake has been implicated in the development of autoimmune diseases (i.e., RA, systemic lupus erythematosus, multiple sclerosis, and Crohn’s disease) [6, 7]. In addition, excessive salt loading promotes pro-inflammatory responses in RA patients by affecting various types of immune cells [5, 6], and dietary salt presents a dose-dependent risk for the emergence of self-reported RA [8]. However, the clinical association between high salt intake and current disease activity of RA is still unclear.

Previous epidemiological studies have used various methods for estimating daily salt intake, which include Tanaka’s formula and Kawasaki’s formula [9, 10]. Tanaka’s formula is commonly used index but requires clinical information about body weight, height, and age as well as urinary Na and Cre concentrations. Recently, clinical evidence has emerged suggesting that the urinary sodium-to-potassium (Na/K) ratio is a simple and useful index of dietary salt loading [11, 12]. The urinary Na/K ratio is just calculated by dividing the measured spot urinary Na and K concentrations and has a stronger correlation with blood pressure (BP) levels than Tanaka’s formula in the general population. However, so far there have been only small-scale studies using the urinary Na/K ratio for evaluation of clinical characteristics in an RA population [13].

In the present study, to determine whether the dietary salt loading is an important factor for current disease activity and hypertension in an RA population, we assessed statistical associations between the urinary Na/K ratio and RA disease activity as well as between the urinary Na/K ratio and hypertension in RA patients.

## Methods

### Study design and participants

We conducted a cross-sectional study of RA patients who participated in the Kyoto University Rheumatoid Arthritis Management Alliance cohort (KURAMA cohort study) [14, 15]. The cohort was founded in May 2011 on the principle of appropriate control and improved prognosis for RA patients at the Center for Rheumatic Diseases in Kyoto University Hospital. A total of 441 RA outpatients who visited the hospital between May 1 and November 30, 2016, and who fulfilled the 2010 American College of Rheumatology (ACR)/European League against Rheumatism (EULAR) classification were included [16]. Of the 441 participants, we excluded those with the following conditions: unsuccessful measurement of clinical parameters related to this study and lack of a complete dataset of body composition (*n* = 70); those with confounding conditions or treatments such as dialysis, hepatitis, sex-hormone replacement or suppression therapy, and psychiatric disorders (*n* = 35) were also excluded. The remaining 336 participants were subjected to the analysis. All study procedures were in accordance with the Declaration Helsinki and were approved by the ethics committee of Kyoto University Graduate School and Faculty of Medicine (Approval number: R0357). In all cases, patient consent was obtained prior to sample and data collection.

### Analysis of urine samples

Spot urine samples were collected and stored at − 80 °C. The concentrations of urinary sodium (Na), potassium (K), and Creatinine (Cre) were measured using Electrolyte Analyzer and enzymatic method, respectively (LSI Medience Co., Tokyo, Japan). The urinary Na/K ratio used was just calculated by dividing the measured spot urinary Na and K concentrations. Estimated daily salt intake was calculated using following Tanaka’s formula, which includes urinary Na, Cre, body weight, height, and age [10]: Daily salt intake (using Tanaka’s formula): 21.98 × {Na (mEq/l) × 24-h Cre excretion/[Cre (mg/dl) × 10]}^0.392^ × 0.0585. 24-h Cre excretion was calculated using following formula: height (cm) × 16.14 + body weight (kg) × 14.89–age× 2.04–2444.45.

### RA-related factors and other clinical parameters

Disease activity and physical disability of RA was assessed using the following parameters: the 28-Joint RA Disease Activity Score (DAS28-ESR) and the health assessment questionnaire-disability index (HAQ). The following laboratory data were also evaluated: C-reactive protein (CRP), serum Creatinine (Cre), estimated glomerular filtration (eGFR), rheumatoid factor (RF), and anti-cyclic citrullinated peptide (anti-CCP antibody). Information on current RA therapeutics including use of methotrexate (MTX), prednisolone (PSL), biological agents, nonsteroidal anti-inflammatory drugs (NSAIDs), and cyclosporin/leflunomide/tacrolimus was extracted from the medical records.

### Definition of hypertension and its related parameters

Branchial blood pressure was measured once after a few minutes rest in the sitting position by automatic digital monitor. Using this measured value of systolic blood pressure (SBP) and diastolic BP (DBP), we defined hypertension by systolic blood pressure (SBP) ≥ 140 mmHg or diastolic blood pressure (DBP) ≥ 90 mmHg, or receiving antihypertensives which were surveyed using a self-reported questionnaire. We also collected data on hypertension-confounding factors including past history of cerebral or cardiovascular disease (*n* = 6) and diabetes mellitus (*n* = 29).

### Statistical analysis

To analyze tertiles stratified by urinary Na/K ratio level, a Cochran-Armitage trend test for categorical variables and a Jonckheere-Terpstra trend test for continuous variables were performed. To determine the relationship between blood pressure and urinary Na/K ratio, urinary Na/K ratio, SBP, and DBP were compared by use of Spearman’s rank correlation coefficient. Multivariate analysis was used to assess the association between the prevalence of hypertension and urinary Na/K ratio. The hypertension state was classified as zero for the absence of hypertension and one as the presence of hypertension. After excluding RA patients with confounding factors including diabetes mellitus and the history of cerebral or cardiovascular disease, we performed a multivariate logistic analysis with adjustment for variables known to be associated with hypertension including sex, age, and smoking status. In addition, a sub-analysis using Fisher’s exact test was performed to examine whether some of cs DMARDs (cyclosporine (*n* = 1)/leflunomide (*n* = 6)/tacrolimus (*n* = 25)), NSAIDs (*n* = 150), and biological agents, which may be associated with hypertension, affected the prevalence of hypertension in this study. To assess the association between RA disease activity and urinary Na/K ratio, multiple linear regression analysis was carried out with adjustment for covariates known to be associated with disease activity including sex, age, RF, anti-CCP antibody, smoking, current therapeutics (use of MTX, PSL and biological agents), eGFR, and BMI [17]. Because PSL use and sex difference may be confounding factors for both disease activity and urinary Na/K ratio, additional multivariate analysis that did not include PSL use or sex difference was performed. Statistical significance was determined by use of JMP 14.0.0 software (SAS Institute Inc., Cary, NC, USA) and SPSS Statistics 26 software (IBM, Armonk, NY, USA); *P* values < 0.05 were considered significant.

## Results

### Characteristics of study participants

The baseline characteristics of the 336 patients with RA are shown in Table 1. The mean age and the average RA duration were 61.8 years and 10.6 years, respectively. Compared to other reports, current RA activity measured by DAS28-ESR was generally low, possibly due to intensive treatments including biologic agents [18]. Indicators of salt intake including urinary Na/K ratio and estimated daily salt intake were similar compared to those in other reports [8, 13]. MTX, biological agent, and PSL were used in 73.2%, 51.8%, and 20.8% of RA patients, respectively.

**Table 1:** Clinical characteristics of study population (*n* = 336)

Age, years	61.8 ± 12.0
Male, *n* (%)	57 (17.0)
BMI, kg/m^2^	22.7 ± 3.7
Smoking status, *n* (%)	28 (8.3)
Diabetes mellitus, *n* (%)	29 (8.6)
Cerebral or cardiovascular disease, *n* (%)	6 (1.8)
RA-related parameters	
Duration, years	10.6 ± 9.6
RF, IU/mL	38.5 (0–2833.6)
Anti-CCP antibody, U/mL	50.45 (0.6–3260)
CRP, mg/dL	0.1 (0.1–9.6)
DAS28-ESR	2.4 (0.78–7.20)
HAQ score	0.25 (0–2.50)
Laboratory data	
Serum Cre, mg/dL	0.69 ± 0.20
eGFR, ml/min/1.73m^2^	74.6 ± 18.0
Blood pressure	
SBP (Branchial), mmHg	122.9 ± 17.4
DBP (Branchial), mmHg	70.5 ± 11.8
Hypertension, *n* (%)	110 (32.7)
Urinalysis	
Cre, mg/dL	93.9 ± 63.9
Na, mEq/L	102.8 ± 51.7
K, mEq/L	49.9 ± 29.9
Na/K ratio	2.60 ± 1.68
Estimated daily salt intake, g	7.80 ± 2.20
Current RA therapeutics	
MTX use, *n* (%)	246 (73.2)
Other cs DMARDs use, *n* (%)	116 (34.5)
Biological agent use, *n* (%)	174 (51.8)
PSL use, *n* (%)	70 (20.8)

Continuous variables are presented as mean (± standard deviation) and categorical variables are presented as numbers (%). Data on RA-related parameters are expressed as median (range)

*Abbreviations*: *BMI* body mass index, *RF* rheumatoid factor, *anti-CCP antibody* anti-cyclic citrullinated peptide antibody, *CRP* C-reactive protein, *DAS28-ESR* 28-joint disease activity score using erythrocyte sedimentation, *HAQ* health assessment questionnaire, *Cre* creatinine, *eGFR* estimated glomerular filtration, *SBP* systolic blood pressure, *DBP* diastolic blood pressure, *Na* sodium, *K* potassium, *MTX* methotrexate, *csDMARD* conventional synthetic disease modifying anti-rheumatic drugs, *tsDMARD* targeted synthetic DMARD, *PSL* prednisolone

### Comparison of characteristics in urinary Na/K ratio tertiles

To clarify the effect of the Na/K ratio on RA-related and hypertension-related factors, RA patients were stratified into tertiles by Na/K ratio, and characteristics were compared among the three groups. Under this stratification, we confirmed that urinary Na/K ratio well correlated with the estimated salt intake calculated from Tanaka’s formula. As urinary Na/K ratio was increased, the prevalence of hypertension and measured value of blood pressure, both systolic and diastolic, increased (Table 2). The current RA disease activity scores including DAS28-ESR and DAS28-CRP also increased significantly as urinary Na/K ratio increased. Age, BMI, eGFR, and the percentage of males also increased along with the Na/K ratio. Regarding RA therapeutics, as the urinary Na/K ratio increased, the percentage of MTX use decreased while the percentage of PSL use increased.

**Table 2:** Characteristics of RA patients stratified by urinary Na/K ratio

Urine Na/K ratio	Tertile 1	Tertile 2	Tertile 3	
	< 1.71		2.94 <	
(*N* = 336)	*n* = 112 (33.3%)	*n* = 112 (33.3%)	*n* = 112 (33.3%)	***P*** value *
Age, year	60.0 ± 13.2	62.4 ± 11.8	63.1 ± 11.0	0.143
Male sex, *n* (%)	10 (8.92)	22 (19.64)	25 (22.32)	0.001
Body mass index, kg/m^2^	22.0 ± 3.1	22.6 ± 3.7	23.4 ± 4.1	0.011
Smoking habit, *n* (%)	7 (6.25)	11 (9.82)	10 (8.93)	0.547
Daily salt intake (g/day)	6.09 ± 1.36	7.67 ± 1.44	9.66 ± 2.04	< 0.001
Laboratory data				
Serum Cre, mg/dL	0.69 ± 0.16	0.72 ± 0.27	0.66 ± 0.16	0.081
eGFR, ml/min/1.73m^2^	72.9 ± 18.2	73.0 ± 18.3	77.8 ± 17.2	0.01
CRP, mg/dL	0.32 ± 0.73	0.33 ± 0.65	0.45 ± 1.19	0.46
RF, IU/mL	100.3 ± 302.5	142.5 ± 279.4	131.4 ± 289.0	0.246
anti-CCP antibody, U/mL	201.8 ± 435.8	242.8 ± 446.3	223.5 ± 444.2	0.216
MMP-3, ng/mL	73.8 ± 73.7	93.5 ± 86.0	96.3 ± 105.2	0.302
RA disease characteristics				
Disease duration, year	9.66 ± 9.62	10.91 ± 9.82	11.16 ± 9.32	0.117
DAS28-ESR	2.40 ± 0.83	2.53 ± 0.96	2.74 ± 1.08	0.025
DAS28-CRP	1.92 ± 0.73	2.01 ± 0.79	2.22 ± 1.00	0.042
Blood pressure				
SBP (Branchial), mmHg	120.4 ± 16.5	121.2 ± 16.4	126.9 ± 18.5	0.009
DBP (Branchial), mmHg	69.2 ± 10.5	69.3 ± 11.3	72.9 ± 13.1	0.043
Hypertension, *n* (*%)*	25 (22.3)	37 (33.0)	48 (42.9)	0.0011
Current RA therapeutics				
MTX use, *n* (%)	90 (80.3)	80 (71.4)	76 (67.9)	0.035
Biological agent use, *n* (%)	63 (56.3)	55 (49.1)	56 (50.0)	0.386
Prednisolone use, *n* (%)	17 (15.2)	23 (20.5)	30 (26.8)	0.04

Continuous variables are presented as mean (± standard deviation) and categorical variables are presented as numbers (%). Estimated daily salt intake was calculated using Tanaka’s formula

*Abbreviations*: *RA* rheumatoid arthritis, *Cre* creatinine, *eGFR* estimated glomerular filtration, *RF* rheumatoid factor, *anti-CCP antibody* anti-cyclic citrullinated peptide antibody, *DAS28-ESR* 28-joint disease activity score using erythrocyte sedimentation, *CRP* C-reactive protein, *HAQ* health assessment questionnaire, *MTX* methotrexate

*P values are calculated using Cochran-Armitage trend test for categorical variables and Jonckheere-Terpstra trend test for continuous variables

### Urinary Na/K ratio is positively associated with measured blood pressure and prevalence of hypertension

**Fig. 1:**
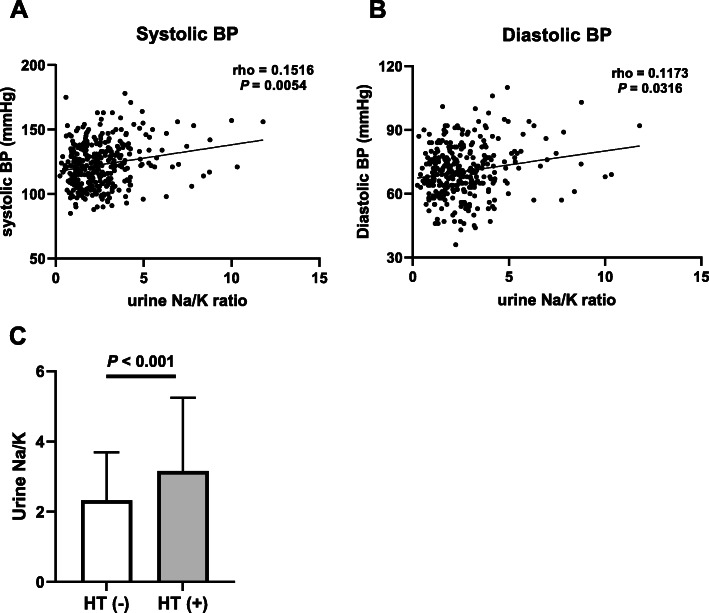
The association between urinary Na/K ratio and blood pressure in RA patients. **a**, **b** Association between urinary Na/K ratio and systolic blood pressure (**a**) and between urinary Na/K ratio and diastolic blood pressure (**b**). **c** Comparison of the spot urine Na/K ratio in the presence or absence of hypertension. *P* values were obtained from the results of Spearman’s rank correlation coefficient (**a**, **b**) and unpaired student *t* test (**c**). Abbreviations: *HT* hypertension, *BP* blood pressure, *RA* rheumatoid arthritis

Although urinary Na/K ratio is a well-known indicator of blood pressure levels in the general population [11, 12], whether this is true in RA patients is not known. The relationship between urinary Na/K ratio and measured blood pressure was therefore examined in RA patients. A significant positive association between urinary Na/K ratio and systolic blood pressure (rho = 0.1516, *p* = 0.0054) as well as between urinary Na/K ratio and diastolic blood pressure (rho = 0.1173, *p* = 0.0316) was observed. (Fig. 1a, b). Furthermore, the urinary Na/K ratio of RA patients with hypertension was higher than that of RA patients without hypertension (Fig. 1c), and multivariate logistic analysis after adjustment for sex, age, and smoking showed an independent and positive association between urinary Na/K ratio and prevalence of hypertension in RA patients (OR 1.34, 95% CI 1.13–1.57, *p* <  0.001) (Table 3). A sub-analysis revealed that the use of a part of cs DMARDs (cyclosporin/leflunomide/tacrolimus) (Supplementary Figure S1A), NSAIDs (Supplementary Figure S1B), and bio DMARDs (Supplementary Figure S1C) were not significantly associated with the prevalence of hypertension. These findings strongly suggest that urinary Na/K ratio is an indicator of hypertension in RA patients as well as in the general population.

**Table 3:** Multivariate logistic analysis for the factors associated with hypertension

Variables	OR	95% CI	***P*** value
Age (1 year)	1.09	1.08–1.13	< 0.001
Urinary Na/K ratio (1)	1.34	1.13–1.57	< 0.001
Sex (male = 1, female = 0)	1.56	0.70–3.47	0.27
Current smoking (+)	0.71	0.20–2.48	0.59

Results of multivariate logistic regression regarding the presence of hypertension in RA patients. We constructed dummy variables as follows: 0 = without hypertension and 1 = with hypertension, and logistic analysis was carried out with potential confounders including age, sex, and current smoking status

*Abbreviations*: *RA* rheumatoid arthritis, *OR* odds ratio

### Urinary Na/K ratio is independently associated with current RA disease activity

To determine whether urinary Na/K ratio contributes to current RA disease activity, we performed multiple regression analysis with DAS28-ESR as the dependent variable. After adjustment for covariates known to be related to disease activity, urinary Na/K ratio was found to be independently and positively associated with DAS28-ESR (estimate 0.12, *p* <  0.001) (Table 4). In addition, because the use of PSL and sex difference may be a confounding factor affecting both disease activity and urinary Na/K ratio [19], subgroup analysis was performed to account for use of PSL and sex difference. Urinary Na/K ratio remained independently associated with DAS28-ESR in gender-separated analysis (Supplementary Table S1) as well as in PSL-separated analysis (Supplementary Table S2). These results indicate that urinary Na/K ratio is an independent indicator-associated with current RA disease activity.

**Table 4:** Multivariate analysis for independent factors associated with DAS28-ESR

Dependent variables	Independent variables	Estimates	Std. Error	95%CI		***P*** value
				Lower	Upper	
DAS28-ESR	Sex (male)	−0.58	0.14	− 0.85	− 0.31	< 0.0001
	Prednisolone (+)	0.48	0.12	0.23	0.72	0.0001
	Urinary Na/K ratio	0.11	0.030	0.048	0.170	0.0004
	Age (1 year)	0.0150	0.0046	0.0055	0.024	0.0016
	RF (1 IU/mL)	0.00054	0.00019	0.00017	0.00091	0.0042
	Biological agent (+)	−0.23	0.098	−0.42	−0.033	0.021
	Anti-CCP antibody (10 U/mL)	0.00	0.00	0.00015	0.0048	0.037
	BMI	−0.024	0.014	−0.050	0.0029	0.081
	eGFR (1 ml/min/1.73m^2^)	0.0051	0.0030	−0.00084	0.011	0.092
	Smoking (+)	−0.18	0.19	−0.54	0.19	0.34
	MTX (+)	−0.0058	0.12	−0.23	0.22	0.96

Results of multiple regression analysis adjusted for urinary Na/K ratio and other variables including sex, age, RF, anti-CCP antibody, smoking status, current therapeutics (the use of Methotrexate, Prednisolone, and biological agents), eGFR, and BMI

*Abbreviations*: *DAS28-ESR* 28-Joint Disease Activity Score using erythrocyte sedimentation rate, *RF* rheumatoid factor, *anti-CCP antibody* anti-cyclic citrullinated peptide antibody, *BMI* Body mass index, *eGFR* estimated glomerular filtration, *MTX* methotrexate, *CI* confidence interval

## Discussion

In the present study, we show a statistical correlation between RA disease activity and urinary Na/K ratio as well as between hypertension and urinary Na/K ratio. The Na/K ratio of spot urine was positively associated with systolic and diastolic BP and was significantly associated with the prevalence of hypertension even after covariate adjustment. In addition, in multivariate analysis including RA-related factors, urinary Na/K ratio was independently correlated with current disease activity score (DAS28-ESR). These results indicate that urinary Na/K ratio reflects not only hypertension but also current disease status in RA patients.

Our finding of a statistical association between urinary Na/K ratio and hypertension in RA patients corresponds to previous findings in the general population [11, 12, 19]. Abundant evidence has recently emerged indicating that both excess sodium and potassium deficit participate in the development of hypertension [20–22] and that the combined effect of higher sodium and lower potassium levels on BP is greater than that of either one alone [11, 23]. Similarly, the urinary Na/K ratio has a stronger statistical relationship with BP levels than that of either Na or K secretion alone [11, 24], and also associates with left ventricular hypertrophy and cardiovascular disease [25, 26]. In addition, dietary modifications that can reduce the urinary Na/K ratio are recommended as well-established nutritional therapies for hypertension, such as salt restriction and increased potassium intake (i.e., a diet rich in fruits and vegetables) [27–29]. Considering these findings together, the urinary Na/K ratio may be useful as an indicator of hypertension in the RA population as it is in the general population; dietary modification strategies that reduce the urinary Na/K ratio in the general population may well benefit RA patients.

We also show a significant correlation between DAS28-ESR and urinary Na/K ratio. Urinary Na/K ratio is a strong indicator of hypertension and is affected by both dietary salt and potassium as mentioned above. Recently, basic and clinical studies have reported that sodium and potassium are closely related to the immune system and RA development [7, 8, 30]. A high sodium concentration enhances differentiation of potentially pathogenic Th17 cells [31], promotes pro-inflammatory macrophage polarization [32], and reduces anti-inflammatory responses of Treg cells and M2 macrophages [33, 34]. In animal models, mice with collagen-induced arthritis (CIA) on high salt diet show severe joint inflammation that is accelerated by increased Th-17 cell differentiation [35]. In epidemiological studies, dietary sodium has a dose-dependent relationship with the emergence of RA [8], and high salt intake combined with smoking results in increased risk for the appearance of anti-CCP antibodies [36]. In addition, a pilot study has shown that potassium supplementation improves joint pain in RA patients with hypokalemia [37]. Moreover, our group has previously reported that potassium-rich ingredients such as fruits and vegetables are significantly associated with lower RA disease activity [38]. In summary, the urinary Na/K ratio is an independent disease activity marker of RA, and increased Na intake and decreased K intake may contribute to RA pathogenesis.

In multivariate logistic analysis, other variables such as RF, anti-CCP antibody, age, sex, and PSL use were also associated with DAS28-ESR. These results are in accordance with previous reports. High titers of RF or anti-CCP antibody are well-known to be unfavorable prognostic factors in RA [39], and female sex is independently associated with increased ESR levels in RA patients [40]. A long-term use of PSL potentially has multiple adverse effects and is usually limited to patients with few therapeutic options or sustained high disease activity.

The present study combines a large-scale cohort data set with a simplified predictor of hypertension. Among several estimation methods of daily salt intake, sodium excretion of 24-h urinary storage is the most reliable, but is inconvenient for large-scale surveys. The Na/K ratio has recently been spotlighted as a useful indicator of salt loading, as it has a stronger association with BP levels than Tanaka’s formula [12] and the Na/K ratio of spot urine is closely correlated with that of 24-h urine collection [41]. The spot urinary Na/K ratio is thus a more suitable method for large sample size investigation.

There are several limitations in the present study. Our cross-sectional study does not imply causation, and there is a possibility of reverse causality, where RA disease activity alters dietary habits resulting in increased salt intake. The long-term effect of urinary Na/K ratio on hypertension and RA disease activity is also still unknown. As for antihypertensive medications, because information on hypertension was collected by a self-reported questionnaire which only asked whether patients received medications or not, we could not obtain antihypertensive medication including diuretics which may affect the urinary Na/K ratio. Although our multiple regression model was adjusted for variables including the presence of MTX, PSL, and biological agents, some effect of combined therapy on the relationship between urinary Na/K ratio and DAS28-ESR could not be excluded. Furthermore, the amount and sensitivity to salt intake differs by background including genetics and dietary habits [28]. In addition, there could be unknown confounding ingredients or nutrients in the diet of our study population. Finally, we did not adjust for certain clinical and lifestyle factors that might affect urinary Na/K ratio such as fasting time and seasonal variation [12].

## Conclusions

**Fig. 2:**
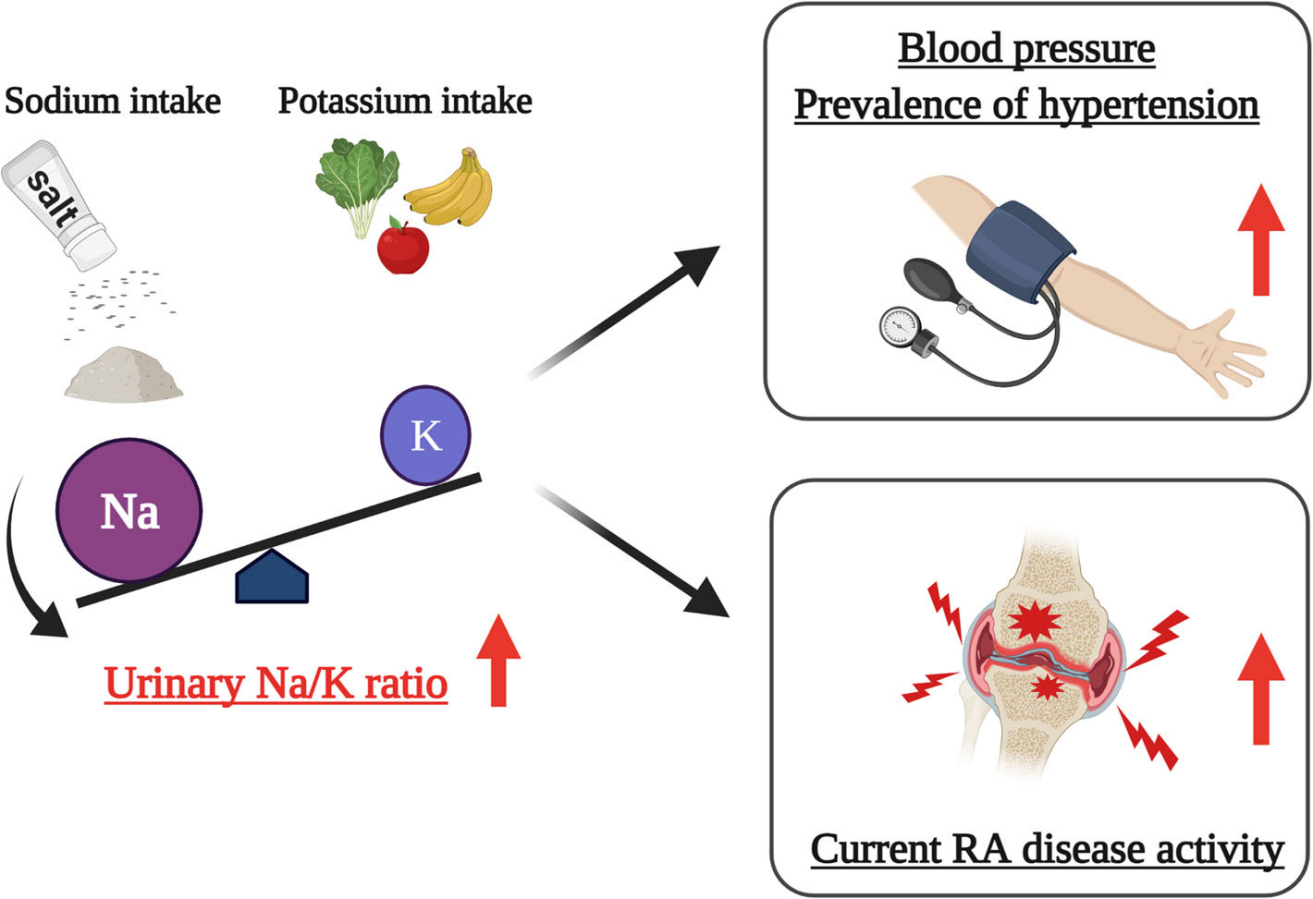
A proposed model of the associations between the urinary Na/K ratio, RA disease activity, and hypertension. An increase in urinary Na/K ratio associated not only with an increase in the prevalence of hypertension, but also with an increase in the disease activity of RA. Nutritional interventions that reduce the urinary Na/K ratio such as salt restriction and potassium supplementation could be potential candidates for attenuating disease activity of RA as well as hypertension. Abbreviations: *Na* sodium, *K* potassium, *RA* rheumatoid arthritis

In summary, this cross-sectional study revealed that the urinary Na/K ratio is significantly associated with RA disease activity as well as prevalence of hypertension. This result raises the possibility that the urinary Na/K ratio is an independent disease activity marker of RA, and increased Na intake and decreased K intake contribute to pathogenesis of RA as well as hypertension (Fig. 2). Thus, nutritional strategies that reduce the urinary Na/K ratio such as salt restriction and potassium supplementation may be candidates for attenuating disease activity of RA as well as hypertension.

## Supplementary Information

**Additional file 1: Supplementary Table 1**. Multivariate analysis for independent factors associated with DAS28-ESR by gender. **Supplementary Table 2**. Multivariate analysis for independent factors associated with DAS28-ESR by the use of PSL.

**Additional file 2: Supplementary Figure 1.** The association between the prevalence of hypertension and RA therapeutics. (A-C) Association between the prevalence of hypertension and the use of a part of cs DMARDs including cyclosporine, leflunomide and tacrolimus (A), NSAIDs (B) and bio DMARDs (C). *P* values were obtained from the results of Fisher’s exact test. Abbreviations: *RA* rheumatoid arthritis*, NSAIDs* nonsteroidal anti-inflammatory drugs, *cs DMARDs* conventional synthetic disease-modifying antirheumatic drugs.

## Data Availability

The datasets analyzed during the current study are available from the corresponding author on reasonable request.

## Abbreviations

RA: Rheumatoid arthritis
KURAMA: Kyoto University Rheumatoid Arthritis Management Alliance cohort
Na: Sodium
K: Potassium
DAS28-ESR: 28-Joint disease activity score using erythrocyte sedimentation
BP: Blood pressure
HAQ: Health assessment questionnaire
CRP: C-reactive protein
eGFR: Estimated glomerular filtration
RF: Rheumatoid factor
Anti-CCP antibody: Anti-cyclic citrullinated peptide antibody
MTX: Methotrexate
PSL: Prednisolone
SBP: Systolic blood pressure
DBP: Diastolic blood pressure
BMI: Body mass index

## References

[1] McInnes IB, Schett G (2017). Pathogenetic insights from the treatment of rheumatoid arthritis. Lancet.

[2] Singh JA, Saag KG, Bridges SL, Akl EA, Bannuru RR, Sullivan MC, Vaysbrot E, McNaughton C, Osani M, Shmerling RH, Curtis JR, Furst DE, Parks D, Kavanaugh A, O'Dell J, King C, Leong A, Matteson EL, Schousboe JT, Drevlow B, Ginsberg S, Grober J, St.Clair EW, Tindall E, Miller AS, McAlindon T (2016). 2015 American College of Rheumatology Guideline for the treatment of rheumatoid arthritis. Arthritis Care Res (Hoboken).

[3] Smolen JS, Landewe R, Bijlsma J, Burmester G, Chatzidionysiou K, Dougados M (2017). EULAR recommendations for the management of rheumatoid arthritis with synthetic and biological disease-modifying antirheumatic drugs: 2016 update. Ann Rheum Dis.

[4] Malmstrom V, Catrina AI, Klareskog L (2017). The immunopathogenesis of seropositive rheumatoid arthritis: from triggering to targeting. Nat Rev Immunol..

[5] Sharif K, Amital H, Shoenfeld Y (2018). The role of dietary sodium in autoimmune diseases: the salty truth. Autoimmun Rev.

[6] Muller DN, Wilck N, Haase S, Kleinewietfeld M, Linker RA (2019). Sodium in the microenvironment regulates immune responses and tissue homeostasis. Nat Rev Immunol.

[7] Scrivo R, Perricone C, Altobelli A, Castellani C, Tinti L, Conti F, et al. Dietary habits bursting into the complex pathogenesis of autoimmune diseases: the emerging role of salt from experimental and clinical studies. Nutrients. 2019;11(5):1013. 10.3390/nu11051013. 10.3390/nu11051013 PMC6566149 31060286

[8] Salgado E, Bes-Rastrollo M, de Irala J, Carmona L, Gomez-Reino JJ (2015). High sodium intake is associated with self-reported rheumatoid arthritis: a cross sectional and case control analysis within the SUN cohort. Medicine (Baltimore).

[9] Kawano Y, Tsuchihashi T, Matsuura H, Ando K, Fujita T, Ueshima H, Working Group for Dietary Salt Reduction of the Japanese Society of Hypertension (2007). Report of the working group for dietary salt reduction of the Japanese Society of Hypertension: (2) assessment of salt intake in the management of hypertension. Hypertens Res.

[10] Tanaka T, Okamura T, Miura K, Kadowaki T, Ueshima H, Nakagawa H, Hashimoto T (2002). A simple method to estimate populational 24-h urinary sodium and potassium excretion using a casual urine specimen. J Hum Hypertens.

[11] Mente A, O'Donnell MJ, Rangarajan S, McQueen MJ, Poirier P, Wielgosz A (2014). Association of urinary sodium and potassium excretion with blood pressure. N Engl J Med.

[12] Tabara Y, Takahashi Y, Kumagai K, Setoh K, Kawaguchi T, Takahashi M, Muraoka Y, Tsujikawa A, Gotoh N, Terao C, Yamada R, Kosugi S, Sekine A, Yoshimura N, Nakayama T, Matsuda F, Nagahama study group (2015). Descriptive epidemiology of spot urine sodium-to-potassium ratio clarified close relationship with blood pressure level: the Nagahama study. J Hypertens.

[13] Carranza-Leon D, Octaria R, Ormseth MJ, Oeser A, Solus JF, Zhang Y, Okafor CR, Titze J, Michael Stein C, Chung CP (2018). Association between urinary sodium and potassium excretion and blood pressure and inflammation in patients with rheumatoid arthritis. Clin Rheumatol.

[14] Minamino H, Katsushima M, Yoshida T, Hashimoto M, Fujita Y, Shirakashi M, Yamamoto W, Murakami K, Murata K, Nishitani K, Tanaka M, Ito H, Inagaki N, Matsuda S (2020). Increased circulating adiponectin is an independent disease activity marker in patients with rheumatoid arthritis: a cross-sectional study using the KURAMA database. Plos One.

[15] Murata K, Ito H, Hashimoto M, Nishitani K, Murakami K, Tanaka M, Yamamoto W, Mimori T, Matsuda S (2019). Elderly onset of early rheumatoid arthritis is a risk factor for bone erosions, refractory to treatment: KURAMA cohort. Int J Rheum Dis.

[16] Aletaha D, Neogi T, Silman AJ, Funovits J, Felson DT, Bingham CO (2010). 2010 rheumatoid arthritis classification criteria: an American College of Rheumatology/European League Against Rheumatism collaborative initiative. Arthritis Rheum.

[17] Masdottir B, Jonsson T, Manfredsdottir V, Vikingsson A, Brekkan A, Valdimarsson H (2000). Smoking, rheumatoid factor isotypes and severity of rheumatoid arthritis. Rheumatology (Oxford).

[18] Giles JT, Allison M, Bingham CO, Scott WM, Bathon JM (2009). Adiponectin is a mediator of the inverse association of adiposity with radiographic damage in rheumatoid arthritis. Arthritis Rheum.

[19] Hedayati SS, Minhajuddin AT, Ijaz A, Moe OW, Elsayed EF, Reilly RF, Huang CL (2012). Association of urinary sodium/potassium ratio with blood pressure: sex and racial differences. Clin J Am Soc Nephrol.

[20] Adrogue HJ, Madias NE (2007). Sodium and potassium in the pathogenesis of hypertension. N Engl J Med.

[21] Fujiwara N, Osanai T, Kamada T, Katoh T, Takahashi K, Okumura K (2000). Study on the relationship between plasma nitrite and nitrate level and salt sensitivity in human hypertension: modulation of nitric oxide synthesis by salt intake. Circulation..

[22] Whelton PK, He J, Cutler JA, Brancati FL, Appel LJ, Follmann D, Klag MJ (1997). Effects of oral potassium on blood pressure. Meta-analysis of randomized controlled clinical trials. JAMA..

[23] Morris RC, Sebastian A, Forman A, Tanaka M, Schmidlin O (1999). Normotensive salt sensitivity: effects of race and dietary potassium. Hypertension..

[24] Group ICR. Intersalt: an international study of electrolyte excretion and blood pressure. Results for 24 hour urinary sodium and potassium excretion. Intersalt Cooperative Research Group BMJ. 1988;297(6644):319–328. 10.1136/bmj.297.6644.319 PMC1834069 3416162

[25] Cook NR, Obarzanek E, Cutler JA, Buring JE, Rexrode KM, Kumanyika SK, Appel LJ, Whelton PK, Trials of Hypertension Prevention Collaborative Research Group (2009). Joint effects of sodium and potassium intake on subsequent cardiovascular disease: the trials of hypertension prevention follow-up study. Arch Intern Med.

[26] Rodriguez CJ, Bibbins-Domingo K, Jin Z, Daviglus ML, Goff DC, Jacobs DR (2011). Association of sodium and potassium intake with left ventricular mass: coronary artery risk development in young adults. Hypertension..

[27] Appel LJ, Brands MW, Daniels SR, Karanja N, Elmer PJ, Sacks FM, American Heart Association (2006). Dietary approaches to prevent and treat hypertension: a scientific statement from the American Heart Association. Hypertension..

[28] Sacks FM, Svetkey LP, Vollmer WM, Appel LJ, Bray GA, Harsha D, Obarzanek E, Conlin PR, Miller ER, Simons-Morton DG, Karanja N, Lin PH, Aickin M, Most-Windhauser MM, Moore TJ, Proschan MA, Cutler JA (2001). Effects on blood pressure of reduced dietary sodium and the Dietary Approaches to Stop Hypertension (DASH) diet. DASH-Sodium Collaborative Research Group. N Engl J Med.

[29] Whelton PK, Appel LJ, Espeland MA, Applegate WB, Ettinger WH, Kostis JB (1998). Sodium reduction and weight loss in the treatment of hypertension in older persons: a randomized controlled trial of nonpharmacologic interventions in the elderly (TONE). TONE Collaborative Research Group. JAMA.

[30] Sundstrom B, Johansson I, Rantapaa-Dahlqvist S (2015). Interaction between dietary sodium and smoking increases the risk for rheumatoid arthritis: results from a nested case-control study. Rheumatology (Oxford).

[31] Kleinewietfeld M, Manzel A, Titze J, Kvakan H, Yosef N, Linker RA, Muller DN, Hafler DA (2013). Sodium chloride drives autoimmune disease by the induction of pathogenic TH17 cells. Nature..

[32] Hucke S, Eschborn M, Liebmann M, Herold M, Freise N, Engbers A, Ehling P, Meuth SG, Roth J, Kuhlmann T, Wiendl H, Klotz L (2016). Sodium chloride promotes pro-inflammatory macrophage polarization thereby aggravating CNS autoimmunity. J Autoimmun.

[33] Binger KJ, Gebhardt M, Heinig M, Rintisch C, Schroeder A, Neuhofer W, Hilgers K, Manzel A, Schwartz C, Kleinewietfeld M, Voelkl J, Schatz V, Linker RA, Lang F, Voehringer D, Wright MD, Hubner N, Dechend R, Jantsch J, Titze J, Müller DN (2015). High salt reduces the activation of IL-4- and IL-13-stimulated macrophages. J Clin Invest.

[34] Hernandez AL, Kitz A, Wu C, Lowther DE, Rodriguez DM, Vudattu N, Deng S, Herold KC, Kuchroo VK, Kleinewietfeld M, Hafler DA (2015). Sodium chloride inhibits the suppressive function of FOXP3+ regulatory T cells. J Clin Invest.

[35] Jung SM, Kim Y, Kim J, Jung H, Yi H, Rim YA, Park N, Kwok SK, Park SH, Ju JH (2019). Sodium chloride aggravates arthritis via Th17 polarization. Yonsei Med J.

[36] Jiang X, Sundstrom B, Alfredsson L, Klareskog L, Rantapaa-Dahlqvist S, Bengtsson C (2016). High sodium chloride consumption enhances the effects of smoking but does not interact with SGK1 polymorphisms in the development of ACPA-positive status in patients with RA. Ann Rheum Dis.

[37] Rastmanesh R, Abargouei AS, Shadman Z, Ebrahimi AA, Weber CE (2008). A pilot study of potassium supplementation in the treatment of hypokalemic patients with rheumatoid arthritis: a randomized, double-blinded, placebo-controlled trial. J Pain.

[38] Murakami I, Murakami K, Hashimoto M, Tanaka M, Ito H, Fujii T, Torii M, Ikeda K, Kuwabara A, Tanaka K, Yoshida A, Akizuki S, Nakashima R, Yoshifuji H, Ohmura K, Usui T, Morita S, Mimori T (2020). Intake frequency of vegetables or seafoods negatively correlates with disease activity of rheumatoid arthritis. PLoS One.

[39] Albrecht K, Zink A (2017). Poor prognostic factors guiding treatment decisions in rheumatoid arthritis patients: a review of data from randomized clinical trials and cohort studies. Arthritis Res Ther.

[40] Siemons L, Ten Klooster PM, Vonkeman HE, van Riel PL, Glas CA, van de Laar MA (2014). How age and sex affect the erythrocyte sedimentation rate and C-reactive protein in early rheumatoid arthritis. BMC Musculoskelet Disord.

[41] Iwahori T, Ueshima H, Miyagawa N, Ohgami N, Yamashita H, Ohkubo T, Murakami Y, Shiga T, Miura K (2014). Six random specimens of daytime casual urine on different days are sufficient to estimate daily sodium/potassium ratio in comparison to 7-day 24-h urine collections. Hypertens Res.

