# Supplementary material for: Urinary sodium-to-potassium ratio associates with hypertension and current disease activity in patients with rheumatoid arthritis: a cross-sectional study
Source: Arthritis Res Ther. 2021 Mar 27;23:96. doi: 10.1186/s13075-021-02479-x (PMC8004419; doi:10.1186/s13075-021-02479-x)
Supplement: Supplementary file 1 — Additional file 1: Supplementary Table 1. Multivariate analysis for independent factors associated with DAS28-ESR by gender. Supplementary Table 2. Multivariate analysis for independent factors associated with DAS28-ESR by the use of PSL. [file 13075_2021_2479_MOESM1_ESM.docx]

**Supplementary Table 1. Multivariate analysis for independent factors associated with DAS28-ESR by gender**

| **Female (*n* = 279)** |  |  |  | **95%CI** | |  |
| --- | --- | --- | --- | --- | --- | --- |
| **Dependent variables** | **Independent variables** | **Estimates** | **Std. Error** | **Lower** | **Upper** | ***p-*value** |
| DAS28-ESR | Prednisolone (+) | 0.54 | 0.14 | 0.26 | 0.81 | **0.0001** |
|  | Urinary Na/K ratio | 0.1 | 0.032 | 0.037 | 0.16 | **0.002** |
|  | RF (1 IU/mL) | 0.00077 | 0.00028 | 0.00022 | 0.0013 | **0.006** |
|  | age (1 year) | 0.013 | 0.0049 | 0.0038 | 0.023 | **0.0064** |
|  | Biological agent (+) | -0.21 | 0.11 | -0.43 | -0.0022 | **0.048** |
|  | eGFR | 0.0061 | 0.0033 | -0.00037 | 0.013 | 0.064 |
|  | Smoking (+) | -0.34 | 0.25 | -0.83 | 0.14 | 0.17 |
|  | Anti-CCP antibody (10 U/mL) | 0.0018 | 0.0014 | -0.00085 | 0.0045 | 0.18 |
|  | BMI | -0.015 | 0.015 | -0.043 | 0.013 | 0.30 |
|  | MTX (+) | -0.0068 | 0.13 | -0.26 | 0.24 | 0.96 |
|  |  |  |  | **95%CI** | |  |
| **Male (n = 57)** | **Independent variables** | **Estimates** | **Std. Error** | **Lower** | **Upper** | ***p-*value** |
|  | BMI | -0.095 | 0.039 | -0.17 | -0.016 | **0.020** |
|  | Urinary Na/K ratio | 0.24 | 0.1 | 0.034 | 0.45 | **0.023** |
|  | Anti-CCP antibody (10 U/mL) | 0.0039 | 0.0026 | -0.0014 | 0.0091 | 0.14 |
|  | eGFR (1 ml/min/1.73m²) | -0.012 | 0.0090 | -0.030 | 0.006 | 0.19 |
|  | Biological agent (+) | -0.26 | 0.24 | -0.75 | 0.22 | 0.28 |
|  | age (1 year) | 0.012 | 0.014 | -0.016 | 0.004 | 0.40 |
|  | RF (1 IU/mL) | 0.00024 | 0.00029 | -0.00035 | 0.00083 | 0.41 |
|  | Prednisolone (+) | 0.25 | 0.30 | -0.36 | 0.85 | 0.42 |
|  | Smoking (+) | 0.19 | 0.29 | -0.40 | 0.78 | 0.51 |
|  | MTX (+) | -0.12 | 0.33 | -0.78 | 0.54 | 0.72 |

**Table S1. Multivariate analysis for independent factors associated with DAS28-ESR by gender**

Results of multiple regression analysis adjusted for urinary Na/K ratio and other variables including age, RF, anti-CCP antibody, smoking status, current therapeutics (the use of MTX, PSL and biological agents), eGFR and BMI in female group (upper) and male group (lower).

Abbreviations: *DAS28-ESR* 28-joint Disease Activity Score using erythrocyte sedimentation rate, *RF* rheumatoid factor, *anti-CCP antibody* anti-cyclic citrullinated peptide antibody, *BMI* Body mass index*, eGFR* estimated glomerular filtration, *MTX* methotrexate*, CI* confidence interval*.*

**Supplementary Table 2.**  **Multivariate analysis for independent factors associated with DAS28-ESR by the use of PSL**

| **PSL non-user (*n* = 266)** |  |  |  | **95%CI** | |  |
| --- | --- | --- | --- | --- | --- | --- |
| **Dependent variables** | **Independent variables** | **Estimates** | **Std. Error** | **Lower** | **Upper** | ***p-*value** |
| DAS28-ESR | RF (1 IU/mL) | 0.0010 | 0.00028 | 0.00045 | 0.002 | **0.00040** |
|  | Sex (male) | -0.50 | 0.15 | -0.79 | -0.20 | **0.0011** |
|  | age (1 year) | 0.015 | 0.0049 | 0.0055 | 0.025 | **0.0022** |
|  | Urinary Na/K ratio | 0.089 | 0.033 | 0.023 | 0.15 | **0.0080** |
|  | Biological agent (+) | -0.25 | 0.100 | -0.46 | -0.05 | **0.015** |
|  | BMI | -0.027 | 0.015 | -0.056 | 0.0014 | 0.062 |
|  | Anti-CCP antibody (10 U/mL) | 0.0024 | 0.0014 | -0.00038 | 0.0051 | 0.090 |
|  | eGFR | 0.050 | 0.0035 | -0.0019 | 0.012 | 0.15 |
|  | Smoking (+) | -0.16 | 0.20 | -0.55 | 0.23 | 0.42 |
|  | MTX (+) | -0.02 | 0.13 | -0.27 | 0.23 | 0.88 |
|  |  |  |  | **95%CI** | |  |
| **PSL user (n = 70)** | **Independent variables** | **Estimates** | **Std. Error** | **Lower** | **Upper** | ***p-*value** |
|  | Urinary Na/K ratio | 0.15 | 0.072 | 0.0091 | 0.30 | **0.038** |
|  | Sex (male) | -0.65 | 0.38 | -1.41 | 0.11 | 0.094 |
|  | Anti-CCP antibody (10 U/mL) | 0.0028 | 0.0026 | -0.0024 | 0.0080 | 0.28 |
|  | RF (1 IU/mL) | 0.00030 | 0.00033 | -0.00036 | 0.00096 | 0.37 |
|  | eGFR (1 ml/min/1.73m²) | 0.0039 | 0.0070 | -0.010 | 0.018 | 0.58 |
|  | BMI | -0.019 | 0.037 | -0.093 | 0.0550 | 0.61 |
|  | Smoking (+) | -0.25 | 0.51 | -1.28 | 0.77 | 0.62 |
|  | age (1 year) | 0.0056 | 0.014 | -0.02 | 0.33 | 0.69 |
|  | Biological agent (+) | -0.090 | 0.28 | -0.65 | 0.47 | 0.75 |
|  | MTX (+) | 0.049 | 0.30 | -0.55 | 0.65 | 0.87 |

**Table S2.**  **Multivariate analysis for independent factors associated with DAS28-ESR by the use of PSL**

Results of multiple regression analysis adjusted for urinary Na/K ratio and other variables including sex, age, RF, anti-CCP antibody, smoking status, current therapeutics (the use of MTX and biological agents), eGFR and BMI in PSL users (upper) and PSL non-users (lower).

Abbreviations: *DAS28-ESR* 28-joint Disease Activity Score using erythrocyte sedimentation rate, *RF* rheumatoid factor, *anti-CCP antibody* anti-cyclic citrullinated peptide antibody, *BMI* Body mass index*, eGFR* estimated glomerular filtration, *MTX* methotrexate*, CI* confidence interval
