# Supplementary figures and images for: Urinary sodium-to-potassium ratio associates with hypertension and current disease activity in patients with rheumatoid arthritis: a cross-sectional study
Source: Arthritis Res Ther. 2021 Mar 27;23:96. doi: 10.1186/s13075-021-02479-x (PMC8004419; doi:10.1186/s13075-021-02479-x)

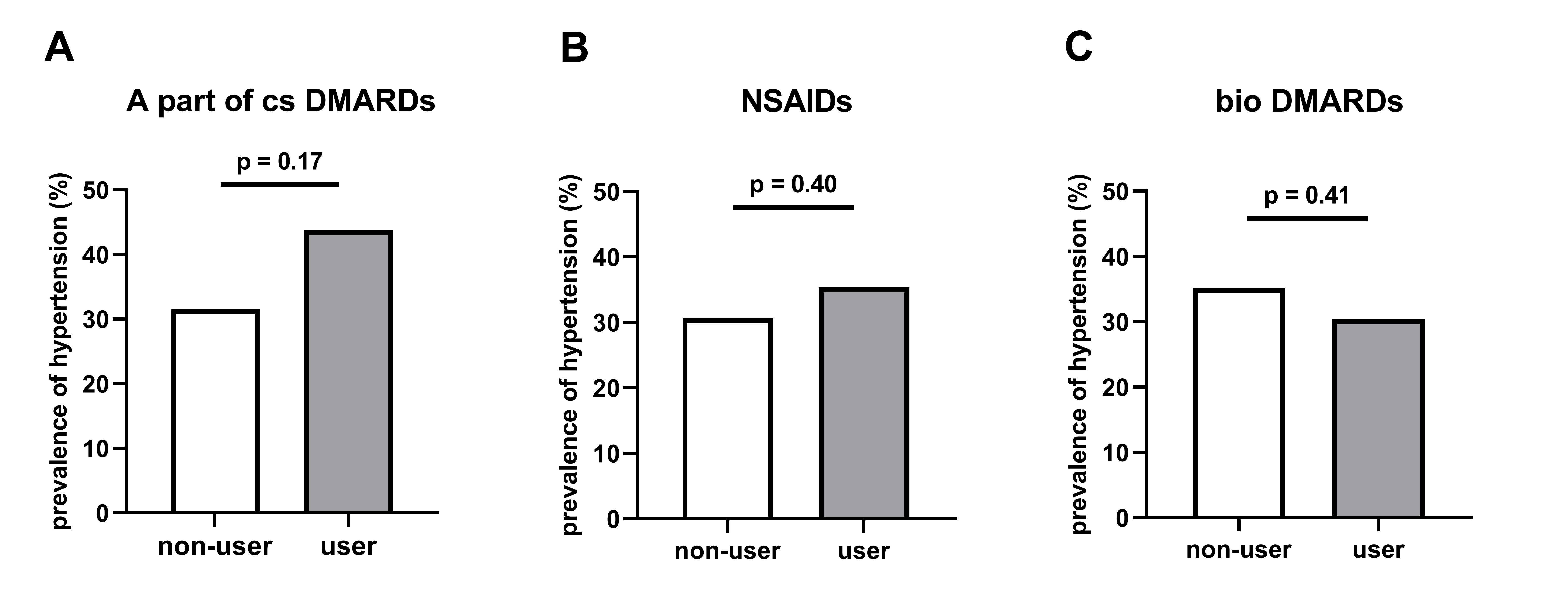

Supplement: Supplementary file 2 — Additional file 2: Supplementary Figure 1. The association between the prevalence of hypertension and RA therapeutics. (A-C) Association between the prevalence of hypertension and the use of a part of cs DMARDs including cyclosporine, leflunomide and tacrolimus (A), NSAIDs (B) and bio DMARDs (C). P values were obtained from the results of Fisher’s exact test. Abbreviations: RA rheumatoid arthritis, NSAIDs nonsteroidal anti-inflammatory drugs, cs DMARDs conventional synthetic disease-modifying antirheumatic drugs. [file 13075_2021_2479_MOESM2_ESM.tif]
